# Classification Efficiency of Pre-Trained Deep CNN Models on Camera Trap Images

**DOI:** 10.3390/jimaging8020020

**Published:** 2022-01-20

**Authors:** Adam Stančić, Vedran Vyroubal, Vedran Slijepčević

**Affiliations:** 1 Department of Engineering, Karlovac University of Applied Sciences, Ivana Meštrovića 10, 47000 Karlovac, Croatia; vedran.vyroubal@vuka.hr; 2 Department of Wildife Management and Nature Protection, Karlovac University of Applied Sciences, Trg J. J. Strossmayera 9, 47000 Karlovac, Croatia; vedran.slijepcevic@vuka.hr

**Keywords:** classification, CNN, efficiency, pre-trained, camera trap

## Abstract

This paper presents the evaluation of 36 convolutional neural network (CNN) models, which were trained on the same dataset (ImageNet). The aim of this research was to evaluate the performance of pre-trained models on the binary classification of images in a “real-world” application. The classification of wildlife images was the use case, in particular, those of the Eurasian lynx (lat. “Lynx lynx”), which were collected by camera traps in various locations in Croatia. The collected images varied greatly in terms of image quality, while the dataset itself was highly imbalanced in terms of the percentage of images that depicted lynxes.

## 1. Introduction

In the present article, the authors suggest the use of various convolutional neural network models as a tool to help scientists classify images according to their content. All classified images were collected as part of other projects that have studied animal behavior and migration in mountainous and wooded parts of Croatia, Gorski Kotar (https://en.wikipedia.org/wiki/Gorski_Kotar, accessed on 25 September 2021), Risnjak (https://en.wikipedia.org/wiki/Risnjak, accessed on 25 September 2021), and Lika (https://en.wikipedia.org/wiki/Lika, accessed on 25 September 2021). The initial purpose of this paper was to help our colleagues of the Wildlife and Environmental/Nature Protection Department at our institution speed up the analysis and classification of the massive number of camera trap images collected. One of the projects focused on the exploration of lynx behavior, habits, and migration and monitoring the number of individual animals in the population. The number of collected images depicting lynxes was extremely low due to the fact that the lynx is an endangered species with a very small population.

It is important to emphasize that all the CNN models used were pre-trained on the same set of images and that each model can be downloaded, while no additional retraining of the models with images collected in the field was conducted. The CNN models described in this paper have different architectures, numbers of parameters, and complexities, which influence their classification rate and accuracy.

This paper is structured as follows: In the Introduction Section, we describe the problem of the classification of the images collected by the camera traps and the phases of the research. The second section describes the questions this paper aimed to address. The third section presents the properties of the various CNN architectures used in the process of image classification. The fourth section compares the machine learning frameworks and describes the chosen framework. The fifth section describes the properties of 36 different pre-trained classification models, the collected images, and the evaluation metrics. The results of the research are presented in the sixth section. The research was conducted in five phases: the collection of the images, the selection of the image classification models, the pre-processing of the collected images for the classification process, the classification of the images, and the analysis of the results. In the image collection phase, the camera traps were installed at various locations where the animals are known to gather. The selection of the appropriate locations was conducted by an expert wildlife preservation team. The camera traps collected both still images and videos. They collected images triggered by either movement in their field of view or by a timer. The process of image collection resulted in a dataset of almost 300,000 images (which varied in size, quality, and content).

The criteria for the selection of classification models were that the selected model be compatible with TensorFlow 1.x, that it uses the TensorFlow-Slim library, be pre-trained on the ImageNet dataset, and be publicly available. All selected models were optimized and “frozen” to improve their inference rate. The process of the optimization and “freezing” of models did not affect the model classification accuracy.

In the pre-processing phase, images were extracted from the video files, with a frequency of two images per second. The extracted images were added to the collection of the captured still images. Duplicate and unsuitable (damaged) files were excluded from the image dataset. All collected images were labeled according to the camera’s location, designation, and timestamp. The last step in the pre-processing phase was the resizing of all collected images, according to the default input size of the particular model.

Every image in the collected dataset was processed with all 36 selected classification models, and the results were stored in a database for later analysis.

The evaluation of the classification models, using a range of different metrics, was performed exclusively on a local computer. It is important to emphasize that all evaluated models were pre-trained on the ImageNet dataset, but in this research, the classification efficiency was evaluated with “real-world” camera trap images.

### Aim of the Research

In the binary classification process conducted with two class labels (“*lynx*”, “*no lynx*”), we focused on finding the answers to the following questions:

- How successful are heterogeneous CNN models at classifying images according to their content?
- What is the CNN model’s efficiency based on the different evaluation metrics?
- Is there a correlation between the model’s complexity, accuracy, and inference rate?
- Can multi-model ensembles of three, four, and five of the top-performing classification models perform better than the best-performing standalone classification model?

## 2. Image Classification

According to LeCun et al. [1], conventional machine-learning methods are limited in their ability to process natural data in their original raw form—in this case, image pixel intensity values captured for each color channel are presented as three two-dimensional arrays. The authors stated that deep-learning methods are suitable for the extraction of the image properties that are important for image classification and object detection tasks. Deep-learning methods (CNN with three or more layers, deep CNN [1]) can learn very complex functions by using groups of nonlinear modules that transform the input data (starting with a captured image) to a higher, more abstract level [1]. The CNN is the most-used architecture in image classification models [2]. Through the use of different types of layers (convolution, pooling, fully connected, etc.), activation functions, computational techniques, and the hyperparameters’ setup, convolutional neural networks are able to extract the image features needed for the classification [3,4]. The process of predicting what object is in an image with the calculated confidence score is called image classification. In order to correctly predict an object in the image, a classification model must be constructed, trained, and evaluated. In the present research, several CNN architectures were used: AlexNet, DenseNet, Inception, MobileNet, NASNet, PNASNet, VGG, and Xception; these architectures are further discussed in the following sections. Training is the process of learning, or simply “teaching” the model how to classify objects in an image. Depending on CNN architecture and the number of images used for training, the process can be very demanding and require significant computation, memory, and storage resources. The same CNN models can be trained using different learning parameters (i.e., the metrics, loss function, optimizer, etc.) and training hyperparameters (i.e., the set values for the learning rate, epoch, batch size, early stopping, etc.) in order to find a suitable model, which makes the training process even more time consuming. It is worth mentioning that dataset images must be processed into a suitable data format before training or inference process, which can also be time consuming, especially with a large number of images. In the present research, all models were trained with the identical set of images (dataset) labeled and divided into 1000 different classes. In this research, we used an image dataset called ImageNet [5,6]. The dataset is also divided into two subsets—namely, the training set, which is used only for model training (and training validation), and test set, which is used only for the classification accuracy evaluation of the completely trained model [5,6]. It should be noted that classification can neither detect multiple objects nor their locations in the examined image; rather, it will output the probabilities of the image representing each of the labels it was trained on.

## 3. Machine Learning Framework

In order to prepare the input data, train, evaluate, and use the image classification model, we needed to use some sort of tools, library, or interface called machine learning (ML) framework. There are many different ML framework solutions on the market such as Amazon ML [7], Google TensorFlow [8], Microsoft Cognitive Toolkit [9], Facebook PyTorch [10], Apache MXNet [11], Theano [12], Berkeley AI Research Caffe [13], etc. In the present research, 36 different CNN models were pre-trained and stored in TensorFlow ML framework checkpoint files. Models were pre-trained with the ImageNet, ILSVRC-2012-CLS [14] image classification dataset. ImageNet consists of 1,200,000 images in the training set and 50,000 in the test set divided into 1000 object categories [14]. The pre-trained model can be additionally retrained with another dataset that has a different distribution of classes. Transfer learning (or domain adaptation) is a technique in which only newly added layers are optimized, while weights (and biases) of the original (pre-trained) model are kept unchanged [15]. In the second technique, called fine-tuning, both weights (and biases) of the newly added classification layers and some of the layers of the original (pre-trained) model are optimized in a retraining process [15].

TensorFlow is an open-source framework suited for numerical computations and large-scale machine learning created by the Google Brain team for deep neural networks (DNNs) [8,16]. The TensorFlow core is written in high-performance C++, while TensorFlow has Application Programming Interfaces (APIs) available in several languages: Python, Julia, JavaScript, C++, Java, Go, and Swift. Google highlights that Python API is (at present) the easiest and most complete, so the entire presented research herein was made with Python and several auxiliary libraries. TensorFlow applications are divided into the two parts: computational graph definition and graph execution. The neural network structure is defined in the computational graph—nodes of the graph represent the tensor objects (constants, variables, placeholders, and operations), while network edges represent the data flow (in form of tensors) between computational operations. Graph execution is performed by the usage of the session object, which places graph operations on Central Processing Unit (CPU)), Graphics Processing Unit (GPU)) or Google’s custom-developed application-specific integrated circuits named Tensor Processing Unit (TPU) [17]. Writing the TensorFlow code may become a complex and cumbersome task, so there are some high-level (or abstraction) libraries that run on the top of TensorFlow. Current versions of TensorFlow supports two abstraction libraries—namely, TensorFlow-Slim [18,19] and Keras [20]. Both libraries help the user to construct, train, evaluate, and use neural network models, with only a few lines of code. In this research, TensorFlow-Slim abstraction library was used for all 36 pre-trained models [18,19].

## 4. Image Classification Models

Image classification models can be created and trained from scratch, but in this case, pre-trained models were used, because of the computationally intensive nature of the training process, which can also be prohibitively expensive if application-specific hardware is used in order to speed up the process [17]. Configuration and building TensorFlow from source is a complex task but results in the optimized binaries for local computer hardware configuration. Better TensorFlow out-of-the-box performance results from using high-level APIs [21] in order to use the instructions supported by the target CPU, GPU, or TPU [22]. TensorFlow also supports different strategies for task distribution across multiple nodes [23]. Even if the TensorFlow environment is optimized for specific hardware, the training process can require days or even weeks to complete [18].

Used image classification models are based on different convolution neural network architectures. Some CNN architectures have more variations of the same architecture (e.g., different number of layers, different input image size or computational techniques etc.) such as Inception, ResNet, VGG, MobileNet, NASNet, and DenseNet. A significant number of used models were trained with TensorFlow, while some models were trained in other frameworks such as Caffe or Keras. Each model trained with Caffe or Keras was converted to a suitable TensorFlow format.

AlexNet model [24,25] is based on a deep CNN architecture of the same name [26,27], which was originally trained with the Caffe framework. AlexNet won the ImageNet competition in 2012. It uses features such as Rectified Linear Unit (ReLU)) activation, data augmentation, dropout, and local response normalization, which are standard parts of modern classification neural networks [28]. AlexNet is considered as a predecessor of all modern CNNs. Densely Connected Convolution Network (DenseNet)) models [27,29] are also based on deep CNN architecture [30] and originally were trained with Keras framework. In the present research, DenseNet-121 (k = 32), DenseNet-161 (k = 48), and DenseNet-169 (k = 32) were used in image classification process. The number in the name of the model denotes the number of layers of the DenseNet model, while parameter k denotes the number of feature maps’ growth rates. Some advantages of DenseNet models are reducing the number of parameters, decreasing the vanishing-gradient problem, feature reuse, and concatenation of the feature maps learned by different layers, in order to improve efficiency [31]. Google is the author of the Inception model, which is implemented in several versions: Inception v1 [32], Inception v2 [33], Inception v3 [34], Inception v4 [35], and a hybrid inception model Inception–Resnet [35]. All used Inception based ImageNet pre-trained models were downloaded from TensorFlow Slim image classification library web page [19]. Inception v1 architecture network was introduced in 2014 and won the ImageNet challenge the same year. The authors of the architecture have taken into account the fact that the objects in an image may have different sizes—larger objects take up larger areas, while smaller objects take up smaller regions of the image. The authors proposed the implementation of inception blocks, which splits the input into different parallel paths (or towers), and at the end of the inception block, the outputs of the different paths were concatenated [31]. Inception architecture introduces 1 × 1 convolutions, to reduce the depth for each path, and uses the global average pooling layers instead of fully connected ones. The Inception v2 version (or Inception-BN) uses batch normalization, in order to use much higher learning rates and be more tolerant toward initialization issues. The improved second version also replaces 5 × 5 convolution kernels with two 3 × 3 kernels, which reduces the number of calculations and saves memory. Inception v3 version factorizes convolutions into smaller convolutions and uses efficient grid size reduction, batch normalization in the auxiliary classifiers, and several inception grids [31,34]. Both architectures, Inception v4 and Inception–ResNet, are presented in the same paper. Inception v4 uses “pure inception architecture” and is a more simplified version of the Inception v3 architecture, with more inception blocks. It also introduces reduction blocks, which are used to change the width and height of the grid. Inception–Resnet is hybrid architecture, i.e., residual connection from the ResNet [27,31,36] model is integrated into the convolution network in order to make the network deeper and faster during the training process [35,37]. MobileNet architecture is specifically optimized for mobile and embedded applications in order to meet the resource constraints [27,31]. It uses two simple global hyperparameters that efficiently trade-off between latency and classification or recognition accuracy [38]. There are three versions of the MobileNet architecture: The first version [38] is based on a streamlined architecture that uses depth-wise separable convolutions to build light weight deep CNNs, while the second version [39] additionally implements linear bottlenecks between the layers and shortcut connections between the bottlenecks. The MobileNet ver. 3 is the third version of the MobileNet architecture [40]. This version uses two algorithms in order to construct suitable network architecture for a specific problem—the MnasNet [41] is used to select optimal network configuration, and NetAdapt [42] is used to fine-tune the proposed configuration. MobileNet ver. 3 is more accurate and faster than MobileNet ver. 2, but the authors of the algorithm present only top-1 accuracy, while top-5 accuracy is not mentioned at all. Models are released as MobileNetV3-Large and MobileNetV3-Small versions, which are targeted for high- and low-resource use cases [40]. Both large and small model versions use all advanced properties of MobileNetv3 architecture, while the so-called minimalistic models do not utilize advanced blocks such as 5 × 5 convolutions, squeeze-and-excite units, and hard swish. In our research four MobileNets ver. 1 (Mob_v1_0.25, Mob_v1_0.50, Mob_v1_0.75, and Mob_v1_1.0) [43], six MobileNets ver. 2 (Mob_v2_0.35, Mob_v2_0.50, Mob_v2_0.75, Mob_v2_1.0, Mob_v2_1.3, and Mob_v2_1.4) [44], and four MobileNets ver. 3 (Mob_v3_lrg, Mob_v3_lrgm, Mob_v3_sml, and Mob_v3_smlm) [44] pre-trained models were used, with the same image input size of 224 × 244 pixels. The number besides the model name and version denotes the depth multiplier, which defines the number of channels in each layer—i.e., value 0.5 will halve the number of channels, which cuts the number of computations and effectively speeds up classification process but with lower accuracy. The Neural Architecture Search Network (NASNet) architecture structure was not predefined by authors, but it was searched by the controller Recurrent Neural Network (RNN) [27,45]. Main structure cells (or blocks) were searched on smaller datasets and then transferred to larger datasets. These cells are called normal cell and reduction cell. A normal cell is a convolution cell that returns a feature map of the same dimension, while the reduction cell returns the halved feature map of the dimension. The authors used slightly differently structured normal and reduction cells in the research and introduced three model versions: NASNet-A, NASNet-B, and NASNet-C. In the presented research here, NASNet-A architecture was used in two versions: NasNet large and NASNet mobile [19]. The authors of the Progressive Neural Architecture Search (PNASNet) [46] propose a Sequential Model-Based Optimization (SMBO) strategy instead of reinforcement learning and evolutionary algorithms introduced in (previously mentioned) NASNet network architecture. PNASNet is eight times faster in terms of total compute and up to five times more efficient in the same search space than NASNet [46]. According to the used number of blocks (and complexity), the PNASNet architecture is denoted from PNASNet-1 (low complexity) to PNASNet-5 (high complexity). In the present research, the PNASNet architecture was used in two versions: PNASNet-5 large and PNASNet-5 mobile [19]. The Residual Network (ResNet)) architecture is focused on solving problems with deep CNNs [27,36]—increasing the convolution network depth leads to network accuracy degradation. Network depth property is crucial in order to gain a better model accuracy [32,36,47]. The authors proposed the implementation of the residual block, which consists of two or three sequential convolutional layers and a shortcut connection between the input of the first and the output of the last layer [31]. ResNet models can be used for extremely deep models, but model accuracy decreases, i.e., a 1202-layer network is less accurate than a 110-layer network [36]. The second version of the ResNet architecture introduced the restructured residual block, with the implementation of identity mappings as skip connections and after-addition activation [48]. ResNet v1 models [49] were originally trained with the Caffe framework and converted to TensorFlow format, while ResNet v2 models were trained with TensorFlow. Both ResNet architecture versions in this research were used with 50-, 101-, and 152-layer deep networks (ResNet v1 50/101/152 and ResNet v2 50/101/152) [19]. The pre-trained ResNet v2 models use Inception pre-processing and input image size of 299 × 299 pixels [19]. The authors from Oxford’s Visual Geometry Group (VGG) found that convolution layers with larger filters (one 5 × 5 filter) can be replaced with two convolution layers with smaller 3 × 3 filters (factorized convolution)—the proposed structure requires lower computational capacities and reduced number of parameters [31,50]. The VGG architecture [47] consists of multiple blocks with stacked convolution layers combined with a max-pooling layer and three fully-connected layers; therefore, final VGG models are computationally expensive and memory inefficient. In the present research, two ImageNet pre-trained models were used—namely, VGG-16 (16 layers) and VGG-19 (19 layers) [19]. Both used VGG models were originally trained with Caffe and converted to suitable TensorFlow formats [51]. The Extreme Inception (Xception) architecture involves depth-wise separable convolutions instead of Inception modules and shortcuts between convolution blocks (such as ResNet) [52]. Xception is very similar to Inception v3 [34] but shows better results [53]. The used Xception pre-trained model was converted from Keras framework into the TensorFlow checkpoint file [54].

### 4.1. Pre-Trained Classification Model Properties

The evaluated image classification models, with respect to their properties, structure, and inference speed, are listed in the next two tables. In the first table, in addition to the model name, the abbreviated name is noted in order to distinguish model versions. Each model has default image width and height size in pixels. The pre-trained models have certain top-n classification accuracy—top-1 is the inference of the model with the highest probability with regard to the expected answer, while top-5 is a situation when the expected answer is in the models’ first five inferences with the highest probability. Top-1 and top-5 accuracy values presented in the table refer to the pre-trained models, not to our actual experiments. As stated, some models were not pre-trained with TensorFlow, so original training ML library was noted for those model versions, listed alphabetically by the model name (Table 1).

The structure and complexity of the model varies with each different model and model variant—number and types of the layers, number of the filters, and filter stride affect the number of parameters. The number of filters, used activation functions, and computation techniques affect the calculation speed. TensorFlow saves the trainable CNN models in two ways—as a checkpoint, which captures the exact value of all parameters, and as SavedModel, which, in addition to the checkpoint, includes a serialized description of the computation. SavedModel format is independent of the source code that created the model and makes it suitable for serving or utilizing in other programming languages [55]. The trained model in form of a checkpoint is stored in four files, in order to separate model graph structure (model.ckpt.meta), value of the variables (model.ckpt.data0000-of-0001), index of the variables (model.ckpt.index), and standalone checkpoint information for older versions of the TensorFlow framework (model.ckpt). Checkpoints files and a SavedModel can additionally be processed into the “frozen” and optimized Protocol buffers (protobuf) format [56]. Freezing a model is a process of converting all model graph variables to constants, while optimization is a process of removing all CNN layers that are not necessary for the inference process. Furthermore, the size of the network in memory and on the disc is proportional to the number of parameters, while latency and power usage of the network corresponds to the number of Floating-point Operations (FLOP ) of the model. Instead of FLOP, some authors use a number of Multiplication and Addition (MAdd) operations or a number of Multiply–Accumulates (MACs) [40,57]. In general, one MAC contains one multiplication and one addition operation, which indicates that 1 MAC = 2 FLOP, but some systems can perform fused multiplication and addition in a single step [58], which indicates that in such cases, 1 MAC = 1 FLOP. The complexity of each evaluated model is presented with two values. The first value is the total number of parameters (trainable and untrainable), retrieved from model checkpoint files with a simple inspection script. As mentioned before, checkpoint (ckpt) files were frozen and optimized in order to speed up image classification process. The second presented value is the number of FLOPs calculated from the frozen and optimized model (pb) file with the TensorFlow profiler application [59]. According to some users, the TensorFlow profiler has some issues with its calculation procedure [60]. All model checkpoint files were downloaded from websites and converted to a frozen file on a local computer. Both calculated values are noted in Table 2.

**Figure 1:**
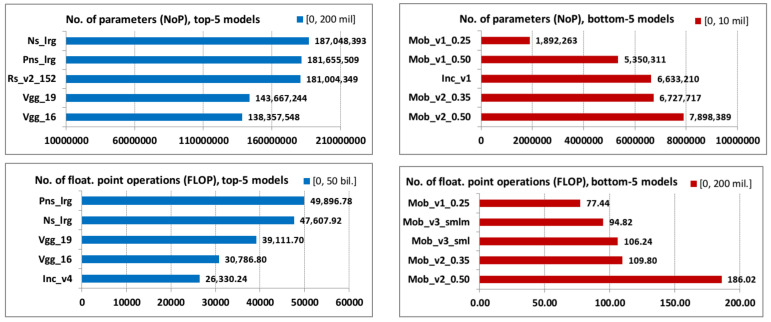
NoP and FLOP top-5 and bottom-5 values.

Top-5 and bottom-5 values of the total number of parameters (NoPs) in checkpoint file and the number of floating-point (FLOP) operations in millions for the frozen graph are displayed in the graphs of Figure 1.

**Figure 2:**
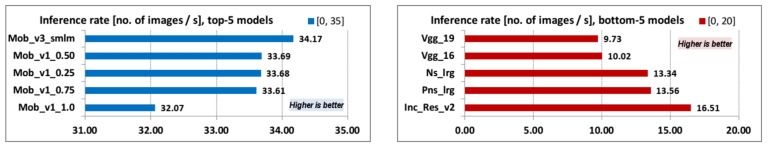
Inference rates; top-5 and bottom-5.

In order to check model inference (or classification) rate, 1000 images were resized to suitable input size, model files were "frozen", and SQLite database [61] was prepared to store top-5 inference results. All images, models, and the database were stored in RAM disk. Model inference speed depends on computer hardware and software configuration—the presented inference speed is the average value of three consecutive measuring processes on (old and cheap) configuration: CPU: AMD A8-6600K APU [62] and GPU: Gigabyte GeForce GTX 1070 8 GB [63]. The inference rate of a particular model is presented in Table 3 and Figure 2.

It is noticeable that CNN architecture complexity influences the models’ inference rate, which confirms the claim that CNN models are a trade-off between inference speed and accuracy—models with faster inference speed results are less accurate and vice versa [43].

There is an additional procedure to speed up the image inference speed on the system, which is using NVIDIA GPU—TensorRT [64,65]. TensorRT will restructure the saved model or the frozen model graph by removing unused output layers and conducts horizontal and vertical layer fusion in order to speed up the inference process. TensorRT also supports different types of calculation precision: 32 FP, 16 FP, and 8 INT, which can additionally improve performance.

### 4.2. Collected Images

As mentioned earlier, the present research was conducted in order to speed up the analysis and classification of a massive number of collected camera trap images. The research of the Eurasian lynx (Lynx lynx) [66] population using photo traps (automatic cameras with IR sensor) was carried out in the period between 2011 and 2019 as a part of two research projects in regions covering an area of about 10,000 km^2^. All photo traps were housed in metal housings at a height of about 40 cm from the ground and were set up in a way that they recorded images the whole time the animal was present in the camera’s field of view. Unfortunately, a camera trap can be triggered by any moving objects such as small or large animals, vegetation in the wind or rain, larger insects and birds, or passing humans and vehicles.

Image quality directly influences the outcome of the classification process, either by humans or machines. Many factors and circumstances have direct impacts on the quality of the image, which can be divided into a few groups—namely, technical properties of the used equipment, location (environment) conditions, and animal behavior. All mentioned properties can have a cumulative effect on the quality of the collected images: the ease of detection of the animal (dependent on the details and textures of the animals and the environment), color palette of the images, clear or vague depiction of an animal, and the deposition of moisture or dirt on the camera lens.

**Figure 3:**
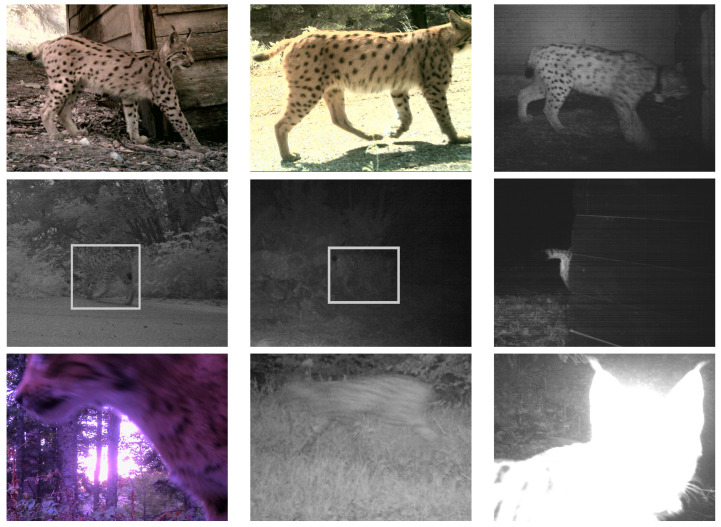
Examples of varying qualities of camera trap images, ranging from very easy to detect to very difficult.

Lynx is a nocturnal and very cautious animal; hence, an extremely low number of high-quality images were captured on the surveilled locations. During the period between 2011 and 2019, camera traps recorded 293,604 images. All images were carefully examined, and lynx was detected on 1630 images (0.55%) by a human operator. It is important to emphasize that 600 images (36.87%) had very low quality and that process of image classification was very demanding even for a human. The following few examples illustrate quality variations of the camera trap images (Figure 3).

The first three images in the first row depict very rare situations when good-quality images were collected in the field. The first two images in the second row are very dark, and it is very hard to detect the lynx (the highlighted rectangle is manually added). The last image in the second row depicts a small part of the lynx. In this case, the characteristic tail of the lynx made recognition possible. The last row depicts some situations where classification process was very difficult; for instance, the lynx was either too close or too fast (motion blur) or the image was overexposed (tuft of black hair on ears is another lynx characteristic).

## 5. Results of the Classification Process

The following tables show the classification results for all evaluated models. All models were utilized in the classification of an identical set of camera trap images. During the classification process, image name, flag whether the image depicts the lynx (detection by human), and the first five classification results were stored in a database. Based on the results of the classification process and the boolean flag whether the lynx was really depicted in the image, basic confusion matrix parameters TP, TN, FP, and FN were calculated. These parameters were used for the calculation of all other derived confusion matrix parameters. Basic confusion matrix values are listed in Table 4 (a detailed description of the parameters can be found in Appendix A.

**Figure 4:**
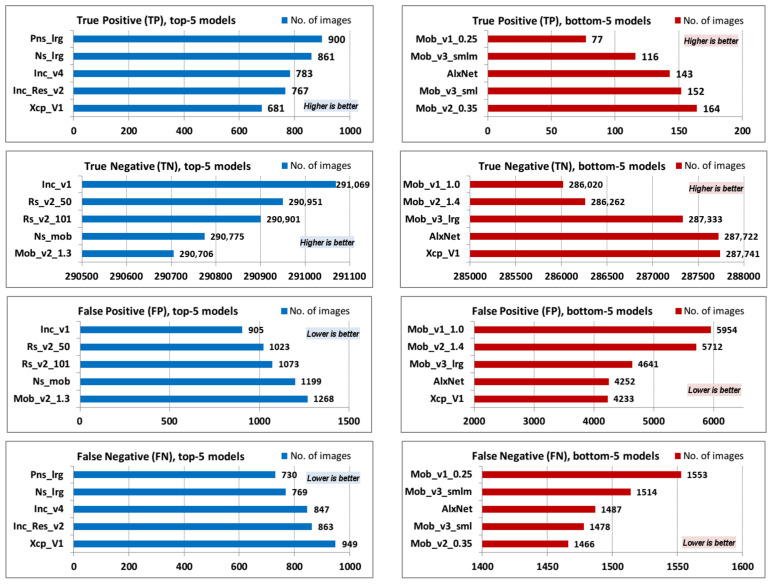
TP, TN, FP, and FN top-5 and bottom-5 values.

Top-5 and bottom-5 values of the confusion matrix basic parameters (TP, TN, FP, and FN) are displayed in Figure 4.

The ImageNet dataset was divided into 1000 categories, but it did not contain category “*lynx*”. There were two ImageNet dataset categories of interest in this research: n02127052 (*lynx, catamount*) and n02125311 (*cougar, puma, catamount, mountain lion, painter, panther, Felis concolor*) [14]. The problem is that in addition to the label *lynx*, there was also a label *catamount*, which reappeared in the second category, so there was an “overlap” of terms. Since these terms are related, we decided to recognize both labels as successful lynx detection in the image. TP results follow the premise that complex models are more accurate and vice versa—models with the highest FLOPs (Pns_lrg) have the highest TP rate, while models with the lowest FLOPs (Mob_v1_0.25) have the lowest TP rate. On the other hand, TN results are quite surprising—the first version of the Inception architecture (Inc_v1) shows the best results, while the first version of the MobileNet architecture with depth multiplier 1.0 (Mob_v1_1.0) shows the worst results. The order of the top and bottom values of the FP parameters follows the order of the top and bottom values of the TN parameters, while the top and bottom values of the FN parameters follows the order of the top and bottom values of the TP parameter.

The following presented parameter values are true-positive rate (TPR or sensitivity), true-negative rate (TNR or specificity), and positive-predictive value (PPV or precision) (Table 5). TPR values are dependent on TP parameter values, so TPR follows the order of the top and bottom values of the TP parameter. TNR values are dependent on TN parameter values, so TNR follows the order of the top and bottom values of the TP parameter. PPV is dependent on TP and FP values and does not follow the order of the top and bottom values of either parameter.

**Figure 5:**
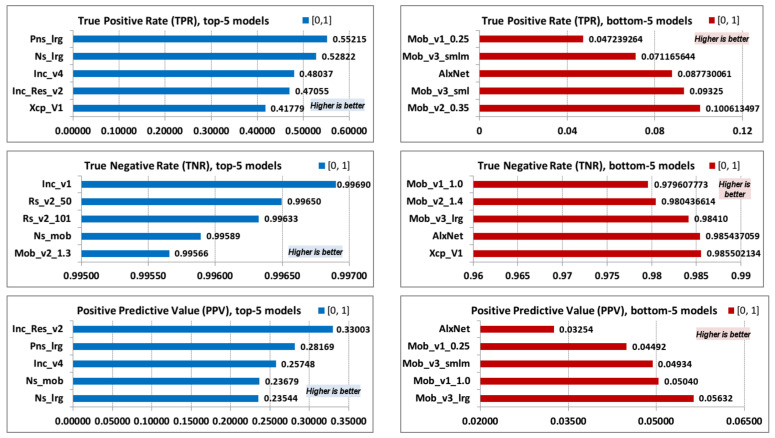
TPR, TNR, and PPV; top-5 and bottom-5.

Top-5 and bottom-5 values of TPR (sensitivity), TNR (specificity), and PPV (precision) are displayed in Figure 5.

The following examined parameters are model accuracy (ACC), error rate (ERR) and balanced accuracy (BAC) (Table 6). Model accuracy is the most famous evaluation parameter, but it can be misleading if the collected image dataset is extremely imbalanced. In the presented case herein, only 1630 images depict the lynx (TP value), and 291,974 images do not depict the lynx (TN value), so high TN value has a significant influence on model accuracy.

**Figure 6:**
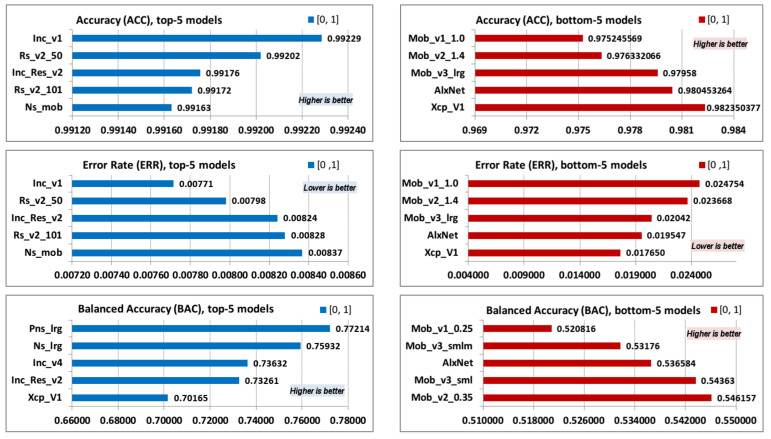
ACC, ERR, and BAC; top-5 and bottom-5 values.

Top-5 and bottom-5 values of models accuracy (ACC), error rate (ERR), and balanced accuracy (BAC) are displayed in Figure 6.

Old and simple Inception version 1 model (Inc_v1) has the highest accuracy of all models. As mentioned before, the reason is that the collected image dataset is extremely imbalanced—only 0.55% of the collected images depict the lynx, while 99.45% do not. As expected, ERR parameter follows the order of the top and bottom values of the ACC parameter. The balanced accuracy (BAC) parameter better presents model accuracy and follows the order of the top and bottom values of the TPR parameter.

The following examined parameters are geometric mean (GM), Youden’s index (YI), and discriminant power (DP) (Table 7). All parameters are dependent on models TPR (or sensitivity) and TPN (or specificity), and they are suitable for imbalanced data.

**Figure 7:**
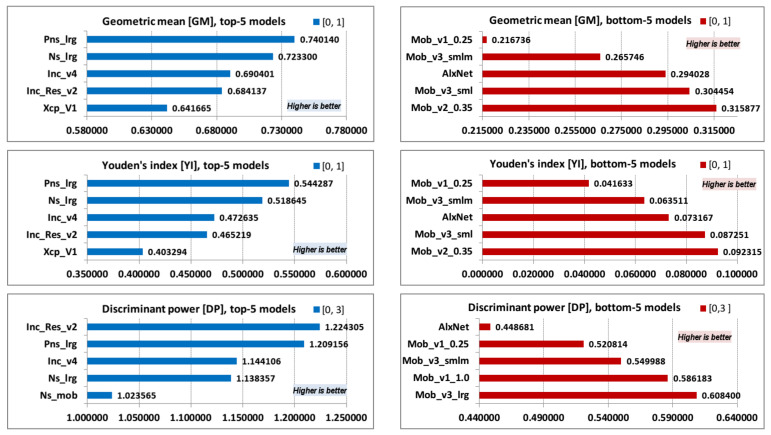
GM, YI, and DP; top-5 and bottom-5 values.

Top-5 and bottom-5 values of geometric mean (GM), Youden’s index (YI), and discriminant power (DP) are displayed in Figure 7.

All three measures are for imbalanced data and depend on models TPR and TNR values. It can be observed that top-5 and bottom-5 orders for GM and YI model values have the same order as the top-5 and bottom-5 TPR values. DP values for top-5 model are listed in almost the same order as PPV values of top-5 models, while DP values of bottom-5 models have exactly the same order as the bottom-5 PPV values.

Finally, the last three classification metric parameters are presented: F1 score (F1), Matthews correlation coefficient (MCC) and Cohen’s kappa (κ) (Table 8). The F1 score does not include TN parameter values, which are very high in our particular case, so MCCs are more suitable parameters in order to evaluate the classification model.

**Figure 8:**
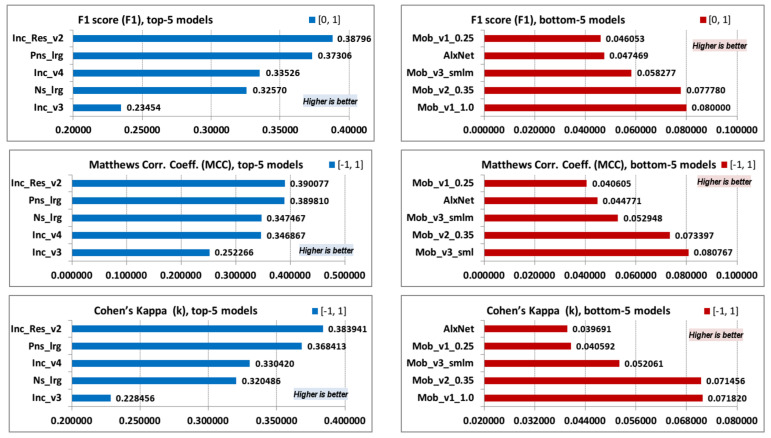
F1, MCC and κ; top-5 and bottom-5 values.

Top-5 and bottom-5 values of models F1 score (F1), Matthew’s correlation coefficient (MCC), and Cohen’s kappa (κ) are displayed in Figure 8.

It can be observed that identical models are listed in the top and the bottom values for F1, MCC, and Cohen’s parameters—even F1 score, which does not imply TN has the same list order as top values. As mentioned earlier, MCC values closer to 1 imply better model prediction capabilities, while values are divided and labeled into several arbitrary ranges. The first four values listed in MCC top-5 values have correlation coefficients between 0.3 and 0.5, which indicates moderate a degree of correlation [67], while the last (Inc_v3) model has a low degree of correlation between real and predicted classification. Cohen’s kappa top-5 values have almost identical list order as the MCC top-5 values. All top-5 parameter values are between 0.2 and 0.4, which indicates a fair agreement between the actual and predicted classification [68,69]. The list of the MCC bottom-5 values are also almost identical and indicate extremely low correlation or a slight agreement between actual and predicted classification.

**Figure 9:**
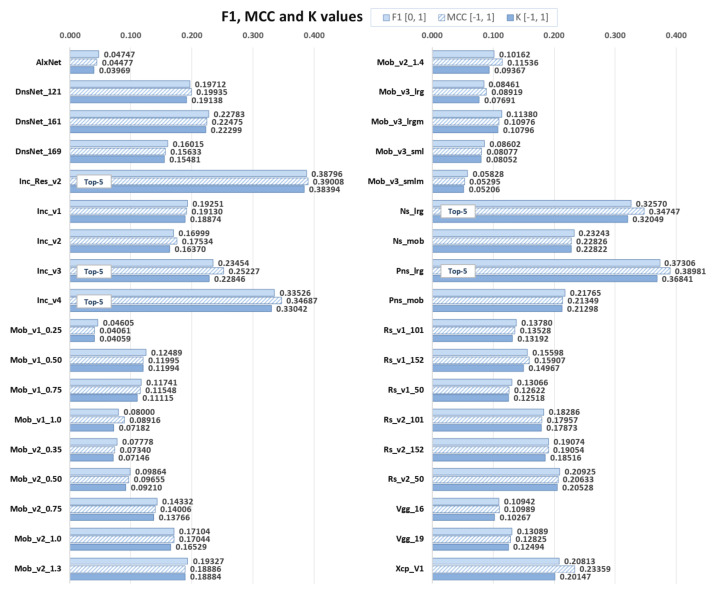
Values of F1 score (F1), Matthew’s correlation coefficient (MCC), and Cohen’s kappa (κ) for all examined pre-trained models for label *lynx*.

Finally F1, MCC, and κ values can be shown for all of the 36 evaluated models. Figure 9 shows how evaluation metrics for selected parameters values of top-5 models (Inc_Res_v2, Pns_lrg, Inc_v4, Ns_lrg, and Inc_v3) are significantly higher than the values of all other models. Unfortunately, all examined models had a poor classification efficiency and, therefore, had limited usability in the present research.

### Properties of Classification Model Ensemble

In this research, only pre-trained classification models were used (as mentioned previously). No additional procedures (e.g., retraining, fine-tuning, transfer learning), which can influence classification accuracy, were performed on any of the models evaluated in this paper. In order to speed up the classification process, all pre-trained models were optimized and "frozen" without repercussions to their accuracy. This research shows that even the best-performing models still have poor classification results. The authors proposed a method of improving the classification accuracy, without additional retraining, by creating an ensemble of a number of best-performing models.

The classification results (i.e., the successful detection of the lynx) were stored in a SQL database, for all of the images in the dataset and all of the pre-trained models. The method of selection of the models to form a model ensemble and the evaluation of such ensemble was automated with a Python script. The Python script calculated TP, TN FP, and FN parameters, based on successful detection of the lynx in the images, for all images in the dataset. These four parameters were the basis for calculating all other metrics in the evaluation of each of the models. Out of 36 evaluated models, only 8 were chosen, based on the cut-off value of kappa being greater than or equal to 0.2 [68,69]. Furthermore, out of the eight models that fit the criterion, the top-five performing ones were chosen, based on the fact that the kappa value sharply decreased by 28.7% between the fourth and fifth best-performing model. To calculate TP, TN, FP, and FN parameters for the model ensemble, an additional parameter was needed—namely, a threshold value that defined the minimum number of models that “had to agree” that the lynx was detected in the image. The threshold parameter could be from 1 to N (all of the models in the ensemble). The optimal threshold number was calculated by brute force evaluation of all combinations of thresholds from 1 to 5.

Top five best-performing classification models were chosen according to the F1, MCC, and Cohen’s kappa values. The chosen models are: Inc_Res_v2, Pns_lrg, Ns_lrg, Inc_v3 and Inc_v4. The classification results from all chosen models were combined into a single “multimodel” or model ensemble for which a new confusion matrix was created. It is worth mentioning that the ensemble was created as a union of standalone pre-trained models and that each model was trained as a standalone model (not as a part of an ensemble). Out of 293,604 collected camera trap images, lynx was manually detected in 1630 images. Furthermore, in order to decide whether an ensemble of pre-trained models detects the lynx, very simple rule was used—i.e., the ensemble detected the lynx only if three or more models reported lynx detection; otherwise, the ensemble did not detect the lynx in the captured image. The ensembles of pre-trained models were named according to the number of individual models; Multi-5 consists of five (Inc_Res_v2, Pns_lrg, Inc_v4, Ns_lrg, and Inc_v3), Multi-4 consists of four (Inc_Res_v2, Pns_lrg, Inc_v4, and Ns_lrg), and Multi-3 consists of three (Inc_Res_v2, Pns_lrg, and Inc_v4) pre-trained models. The confusion matrix for the Multi-5 model is presented in Table 9.

If the ensemble consisted of top-4 models (Inc_Res_v2, Pns_lrg, Ns_lrg, and Inc_v4), then the same rule could be applied—the ensemble detected the lynx only if three individual models reported lynx detection. The confusion matrix for the Multi-4 model is presented in Table 10.

If the ensemble consisted of top-3 models (Inc_Res_v2, Pns_lrg, and Inc_v4), then similar rule could be applied—the ensemble detected the lynx only if all individual models reported lynx detection and vice versa. The confusion matrix for the Multi-3 model is presented in Table 11.

**Figure 10:**
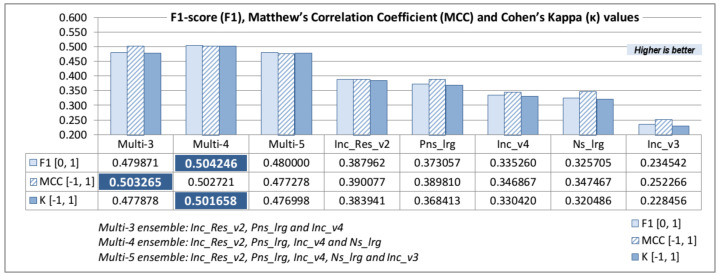
F1, MCC and κ parameter values of the top 5 standalone models and model ensembles.

The comparison of the F1, MCC, and κ parameters values between top-5 standalone models and model ensemble confirms the assumption that the ensemble can reach higher classification efficiency without the need for retraining, as is shown in Figure 10.

F1 score, MCC, and κ maximum values in the data table (Figure 10) are additionally marked and clearly depict how all ensemble models are more efficient than any standalone model. Multi-3 and Multi-5 ensembles have a similar F1 and κ values, while Multi-3 and Multi-4 ensembles have similar MCC values. The Multi-4 ensemble has the best classification metric results, i.e., the highest F1 and κ values and slightly lower than maximum MCC value (Multi-3). The comparison of the best results by model ensembles and the best-performing pre-trained standalone model (Inc_Res_v2) shows that F1 parameter is 23% higher and that κ is 23.5% higher in the case of a Multi-4 ensemble, while MCC is 22.5% higher in the case of a Multi-3 ensemble.

## 6. Conclusions

In this research, a dataset of images collected by camera traps was used. The images depicting the Eurasian lynx in its natural habitat were the focus of this research. Due to the fact that lynx is a nocturnal and very cautious animal, the dataset was imbalanced with regard to the percentage of images depicting the lynx, while images themselves varied greatly in quality. In total, 36 CNN models of different architectures and complexities but trained with the same ImageNet dataset were used for binary classification. Each of the evaluated models was used in the classification process of 293,604 camera trap images, of which only 1630 depicted the lynx. The efficiency of the models was evaluated according to nine distinct evaluation metrics. Based on classification results and information on whether the image depicts the lynx or not (set by a human observer), the confusion matrix and evaluation parameters for each CNN model used were generated.

In order to evaluate CNN model classification efficiency, we focused on three evaluation metric parameters: F1 score (F1), Matthew’s correlation coefficient (MCC), and Cohen’s kappa (κ). This research shows that in the case of a highly imbalanced dataset, Inception–Resnet version 2 (Inc_Res_v2) shows the best results, according to all three (F1, MCC, and κ) evaluation metric parameters. It is surprising that older and less complex model such as Inc_Res_v2 has a minor advantage over Pns_lrg. The next two models in the top-5 list by all evaluation metric parameters are Inception version 4 (Inc_v4) and NasNet large (Ns_lrg). The Ns_lrg model is more complex and slower than Inc_v4. The difference between these two models is more obvious than in the cases of Inc_Res_v2 and Pns_lrg. It is an even greater surprise that the Inception version 3 (Inc_v3) model is listed in top-5 values for F1, MCC, and **κ** evaluation parameters. The observed evaluation parameters values for the Inc_v3 model are significantly lower, compared with the other listed top-5 model values but compared with newer and more complex models (i.e., models with VGG or ResNet architecture), the observed results are better.

Models that showed the worst results in this research were AlexNet and MobileNet version 1 0.25 (Mob_v1_0.25). Other models listed on the bottom-5 evaluation metric parameters list are found in the MobileNet architecture: MobileNet version 1 1.0 (Mob_v1_1.0), MobileNet version 2 0.35 (Mob_v2_0.35), and MobileNet ver. 3 1.0 small minimalistic (Mob_v3_smlm). The bottom-5 results are not so surprising because the AlexNet architecture was presented in 2012, making it the oldest of all evaluated CNN models. In addition, the MobileNet architecture has the simplest model structure, making it more appropriate for systems with low computational and energy resources.

This research showed that the results of the best-performing models are still rather poor, which is in some ways to be expected—used pre-trained deep CNN models were not retrained with additional images collected in the field or altered in any other way. All models were pre-trained with the ImageNet dataset, which proved to be problematic for the classification of images collected by camera traps. The ImageNet dataset consists of clear, high-resolution images, while camera trap images vary in quality and are highly dependent on technical properties of the equipment, location conditions, and animal behavior. High0quality images collected by camera traps are very rare—more of an exception than the rule. To achieve better performances of evaluated models, the user can perform training of a new model from scratch or fine-tuning a model from an existing checkpoint. Regardless of the method chosen (new model or fine-tuning), training (or retraining) of models with “real-world” collected images is necessary in order to gain higher values of the model evaluation parameters.

The assumption that less complex CNN models are less accurate but have higher classification rate (and vice versa is) was proven correct in this research. The listed models with bottom-5 evaluation parameter values were always the models of lower complexity. That does not make them unnecessary and superfluous—to achieve better accuracy, model training with an appropriate dataset is necessary. The less complex model with MobileNet architecture should be trained, tested, and evaluated with location-specific data in order to gain higher accuracy—i.e., models can be trained with collected camera trap images with only two labels, *"lynx"* and *"no lynx"*. Based on the conducted research, it can be assumed that training from scratch of all used (pre-trained) CNN models can improve model classification accuracy and inference rate, no matter how complex they are. This assumption can be verified in future studies.

The next step of the research was to combine the pre-trained models into a multimodel (model ensemble). The motivation for this approach was an assumption that such model ensemble can achieve better classification results, without the need for a costly retraining process. Three model ensembles were created consisting of three, four, and five top-preforming pre-trained models (according to F1, MCC, and κ values). The assumption was proven correct by the results of this research. The Multi-4 ensemble showed significantly better results than the best-performing standalone pre-trained model.

Future studies on the presented subject should be focused on increasing models classification efficiency. The analysis in this study has shown that there were images in which standalone models, as well as model ensembles, failed to detect the lynx, while the animal was detected “manually” by the expert team from the Wildlife and Environmental/Nature Protection Department. This research showed that the pre-trained CNN models are not adequate for the classification of “real-world” images. In order to gain better classification results, each of the examined models should be retrained with a dataset of camera trap image dataset. After the retraining process, models should be carefully reexamined with a new set of images, which are currently being collected by camera traps in various locations.

## Figures and Tables

**Table 1:** List of image classification models pre-trained on ImageNet dataset, model abbreviations, top-1 and top-5 accuracy, and original machine learning library for training.

Model Name and Version	Abbreviation	Top-1	Top-5	ML Library
AlexNet	AlxNet	57.2	80.3	Caffe
DenseNet 121	DnsNet_121	74.9	92.2	Keras
DenseNet 161	DnsNet_161	77.6	93.8	Keras
DenseNet 169	DnsNet_169	76.1	93.1	Keras
Inception-Resnet version 2	Inc_Res_v2	80.4	95.3	Tensorflow
Inception version 1	Inc_v1	69.8	89.6	Tensorflow
Inception version 2	Inc_v2	73.9	91.8	Tensorflow
Inception version 3	Inc_v3	78.0	93.9	Tensorflow
Inception version 4	Inc_v4	80.2	95.2	Tensorflow
MobileNet version 1 0.25	Mob_v1_0.25	49.8	74.2	Tensorflow
MobileNet version 1 0.50	Mob_v1_0.50	63.3	84.9	Tensorflow
MobileNet version 1 0.75	Mob_v1_0.75	68.4	88.2	Tensorflow
MobileNet version 1 1.0	Mob_v1_1.0	70.9	89.9	Tensorflow
MobileNet version 2 0.35	Mob_v2_0.35	60.3	82.9	Tensorflow
MobileNet version 2 0.50	Mob_v2_0.50	65.4	86.4	Tensorflow
MobileNet version 2 0.75	Mob_v2_0.75	69.8	89.6	Tensorflow
MobileNet version 2 1.0	Mob_v2_1.0	71.8	91.0	Tensorflow
MobileNet version 2 1.3	Mob_v2_1.3	74.4	92.1	Tensorflow
MobileNet version 2 1.4	Mob_v2_1.4	75.0	92.5	Tensorflow
MobileNet version 3 1.0 large	Mob_v3_lrg	75.2	N/A	Tensorflow
MobileNet ver. 3 1.0 large mini.	Mob_v3_lrgm	72.3	N/A	Tensorflow
MobileNet version 3 1.0 small	Mob_v3_sml	67.5	N/A	Tensorflow
MobileNet ver. 3 1.0 small mini.	Mob_v3_smlm	61.9	N/A	Tensorflow
NasNet large	Ns_lrg	82,7	96.2	Tensorflow
NasNet mobile	Ns_mob	74.0	91.6	Tensorflow
PnasNet-5 large	Pns_lrg	82.9	96.2	Tensorflow
PnasNet-5 mobile	Pns_mob	74.2	91.9	Tensorflow
ResNet 50 version 1	Rs_v1_50	75.2	92.2	Caffe
ResNet 101 version 1	Rs_v1_101	76.4	92.9	Caffe
ResNet 152 version 1	Rs_v1_152	76.8	93.2	Caffe
ResNet 50 version 2	Rs_v2_50	75.6	92.8	Tensorflow
ResNet 101 version 2	Rs_v2_101	77.0	93.7	Tensorflow
ResNet 152 version 2	Rs_v2_152	77.8	94.1	Tensorflow
VGG 16	Vgg_16	71.5	89.8	Caffe
VGG 19	Vgg_19	71.1	89.8	Caffe
Xception version 1	Xcp_v1	79.0	94.5	Keras

Listed in alphabetical order.

**Table 2:** Image classification models complexity as number of parameters (NoPs) and number of floating-point (FLOP) operations in millions.

Model	NoP	FLOP [mil]	Model	NoP	FLOP [mil]
AlxNet	60,965,224	1450.58	Mob_v2_1.4	24,490,213	1138.56
DnsNet_121	8,062,505	5439.98	Mob_v3_lrg	16,501,740	431.58
DnsNet_161	28,900,937	15,113.90	Mob_v3_lrgm	15,751,077	410.94
DnsNet_169	14,307,881	6491.70	Mob_v3_sml	10,199,749	106.24
Inc_Res_v2	59,244,595	2776.56	Mob_v3_smlm	8,207,269	94.82
Inc_v1	6,633,210	3856.10	Ns_lrg	187,048,393	47,607.92
Inc_v2	11,199,138	11,421.58	Ns_mob	15,483,149	1121.80
Inc_v3	27,182,195	24,529.78	Pns_lrg	181,655,509	49,896.78
Inc_v4	46,074,067	26,330.24	Pns_mob	15,029,139	1168.20
Mob_v1_0.25	1,892,263	77.44	Rs_v1_50	25,610,156	6744.48
Mob_v1_0.50	5,350,311	289.74	Rs_v1_101	44,654,508	14,177.45
Mob_v1_0.75	10,378,151	636.92	Rs_v1_152	60,344,236	21,612.43
Mob_v1_1.0	16,975,783	1118.98	Rs_v2_50	76,802,109	6744.49
Mob_v2_0.35	6,727,717	109.80	Rs_v2_101	133,935,165	14,177.46
Mob_v2_0.50	7,898,389	186.02	Rs_v2_152	181,004,349	21,612.44
Mob_v2_0.75	10,577,461	406.04	Vgg_16	138,357,548	30,786.80
Mob_v2_1.0	14,058,725	584.54	Vgg_19	143,667,244	39,111.70
Mob_v2_1.3	21,598,085	997.98	Xcp_v1	22,910,480	16,731.28

Listed in alphabetical order.

**Table 3:** Classification model image input size and inference rate in number of images processed per second (img/s).

Model	Input Size	img/s	Model	Input Size	img/s
AlxNet	227 × 227	27.79	Mob_v2_1.4	224 × 224	29.66
DnsNet_121	224 × 224	23.04	Mob_v3_lrg	224 × 224	29.37
DnsNet_161	224 × 224	18.66	Mob_v3_lrgm	224 × 224	31.94
DnsNet_169	224 × 224	20.94	Mob_v3_sml	224 × 224	31.56
Inc_Res_v2	224 × 224	16.51	Mob_v3_smlm	224 × 224	34.17
Inc_v1	224 × 224	28.53	Ns_lrg	331 × 331	13.34
Inc_v2	224 × 224	27.77	Ns_mob	224 × 224	22.71
Inc_v3	299 × 299	22.33	Pns_lrg	331 × 331	13.56
Inc_v4	299 × 299	18.65	Pns_mob	224 × 224	22.34
Mob_v1_0.25	224 × 224	33.68	Rs_v1_50	224 × 224	25.15
Mob_v1_0.50	224 × 224	33.69	Rs_v1_101	224 × 224	21.91
Mob_v1_0.75	224 × 224	33.61	Rs_v1_152	224 × 224	19.59
Mob_v1_1.0	224 × 224	32.07	Rs_v2_50	299 × 299	26.09
Mob_v2_0.35	224 × 224	31.15	Rs_v2_101	299 × 299	22.32
Mob_v2_0.50	224 × 224	30.98	Rs_v2_152	299 × 299	19.79
Mob_v2_0.75	224 × 224	31.15	Vgg_16	224 × 224	10.02
Mob_v2_1.0	224 × 224	30.67	Vgg_19	224 × 224	9.73
Mob_v2_1.3	224 × 224	30.22	Xcp_V1	299 × 299	22.67

Listed in alphabetical order: img/s, higher is better.

**Table 4:** Values of the confusion matrix basic parameters true positive (TP), true negative (TN), false positive (FP), and false negative (FN) for label *lynx*.

Model	Number of Images (NoI)				Model	Number of Images (NoI)			
	TP	TN	FP	FN		TP	TN	FP	FN
AlxNet	143	287,722	4252	1487	Mob_v2_1.4	393	286,262	5712	1237
DnsNet_121	445	289,534	2440	1185	Mob_v3_lrg	277	287,333	4641	1353
DnsNet_161	424	290,306	1668	1206	Mob_v3_lrgm	226	289,858	2116	1404
DnsNet_169	303	290,123	1851	1327	Mob_v3_sml	152	290,222	1752	1478
Inc_Res_v2	767	290,417	1557	863	Mob_v3_smlm	116	289,739	2235	1514
Inc_v1	270	291,069	905	1360	Ns_lrg	861	289,178	2796	769
Inc_v2	430	288,975	2999	1200	Ns_mob	372	290,775	1199	1258
Inc_v3	660	288,636	3338	970	Pns_lrg	900	289,679	2295	730
Inc_v4	783	289,716	2258	847	Pns_mob	381	290,484	1490	1249
Mob_v1_0.25	77	290,337	1637	1553	Rs_v1_50	244	290,113	1861	1386
Mob_v1_0.50	206	290,511	1463	1424	Rs_v1_101	288	289,712	2262	1342
Mob_v1_0.75	262	289,403	2571	1368	Rs_v1_152	383	289,076	2898	1247
Mob_v1_1.0	316	286,020	5954	1314	Rs_v2_50	310	290,951	1023	1320
Mob_v2_0.35	164	289,551	2423	1466	Rs_v2_101	272	290,901	1073	1358
Mob_v2_0.50	229	289,190	2784	1401	Rs_v2_152	406	289,753	2221	1224
Mob_v2_0.75	286	289,899	2075	1344	Vgg_16	276	288,835	3139	1354
Mob_v2_1.0	368	289,669	2305	1262	Vgg_19	275	289,677	2297	1355
Mob_v2_1.3	310	290,706	1268	1320	Xcp_V1	681	287,741	4233	949

Listed in alphabetical order: TP, TN, higher is better; FP, FN, lower is better.

**Table 5:** Values of the true-positive rate (TPR), true-negative rate (TNR), and positive-predictive value (PPV) for label *lynx*.

Model	TPR	TNR	PPV	Model	TPR	TNR	PPV
AlxNet	0.08773	0.98544	0.03254	Mob_v2_1.4	0.24110	0.98044	0.06437
DnsNet_121	0.27301	0.99164	0.15425	Mob_v3_lrg	0.16994	0.98410	0.05632
DnsNet_161	0.26012	0.99429	0.20268	Mob_v3_lrgm	0.13865	0.99275	0.09650
DnsNet_169	0.18589	0.99366	0.14067	Mob_v3_sml	0.09325	0.99400	0.07983
Inc_Res_v2	0.47055	0.99467	0.33003	Mob_v3_smlm	0.07116	0.99234	0.04934
Inc_v1	0.16564	0.99690	0.22979	Ns_lrg	0.52822	0.99042	0.23544
Inc_v2	0.26380	0.98973	0.12540	Ns_mob	0.22822	0.99589	0.23679
Inc_v3	0.40491	0.98857	0.16508	Pns_lrg	0.55215	0.99214	0.28169
Inc_v4	0.48037	0.99227	0.25748	Pns_mob	0.23374	0.99490	0.20363
Mob_v1_0.25	0.04724	0.99439	0.04492	Rs_v1_50	0.14969	0.99363	0.11591
Mob_v1_0.50	0.12638	0.99499	0.12343	Rs_v1_101	0.17669	0.99225	0.11294
Mob_v1_0.75	0.16074	0.99119	0.09248	Rs_v1_152	0.23497	0.99007	0.11673
Mob_v1_1.0	0.19387	0.97961	0.05040	Rs_v2_50	0.19018	0.99650	0.23256
Mob_v2_0.35	0.10061	0.99170	0.06339	Rs_v2_101	0.16687	0.99633	0.20223
Mob_v2_0.50	0.14049	0.99046	0.07600	Rs_v2_152	0.24908	0.99239	0.15455
Mob_v2_0.75	0.17546	0.99289	0.12114	Vgg_16	0.16933	0.98925	0.08082
Mob_v2_1.0	0.22577	0.99211	0.13767	Vgg_19	0.16871	0.99213	0.10692
Mob_v2_1.3	0.19018	0.99566	0.19645	Xcp_v1	0.41779	0.98550	0.13858

Listed in alphabetical order: TPR [0,1], TNR [0,1], PPV [0,1]; higher is better.

**Table 6:** Values of model accuracy (ACC), error rate (ERR), and balanced accuracy (BAC) for label *lynx*.

Model	ACC	ERR	BAC	Model	ACC	ERR	BAC
AlxNet	0.98045	0.01955	0.53658	Mob_v2_1.4	0.97633	0.02367	0.61077
DnsNet_121	0.98765	0.01235	0.63232	Mob_v3_lrg	0.97958	0.02042	0.57702
DnsNet_161	0.99021	0.00979	0.62720	Mob_v3_lrgm	0.98801	0.01199	0.56570
DnsNet_169	0.98918	0.01082	0.58977	Mob_v3_sml	0.98900	0.01100	0.54363
Inc_Res_v2	0.99176	0.00824	0.73261	Mob_v3_smlm	0.98723	0.01277	0.53176
Inc_v1	0.99229	0.00771	0.58127	Ns_lrg	0.98786	0.01214	0.75932
Inc_v2	0.98570	0.01430	0.62677	Ns_mob	0.99163	0.00837	0.61206
Inc_v3	0.98533	0.01467	0.69674	Pns_lrg	0.98970	0.01030	0.77214
Inc_v4	0.98942	0.01058	0.73632	Pns_mob	0.99067	0.00933	0.61432
Mob_v1_0.25	0.98914	0.01086	0.52082	Rs_v1_50	0.98894	0.01106	0.57166
Mob_v1_0.50	0.99017	0.00983	0.56068	Rs_v1_101	0.98772	0.01228	0.58447
Mob_v1_0.75	0.98658	0.01342	0.57597	Rs_v1_152	0.98588	0.01412	0.61252
Mob_v1_1.0	0.97525	0.02475	0.58674	Rs_v2_50	0.99202	0.00798	0.59334
Mob_v2_0.35	0.98675	0.01325	0.54616	Rs_v2_101	0.99172	0.00828	0.58160
Mob_v2_0.50	0.98575	0.01425	0.56548	Rs_v2_152	0.98827	0.01173	0.62074
Mob_v2_0.75	0.98836	0.01164	0.58418	Vgg_16	0.98470	0.01530	0.57929
Mob_v2_1.0	0.98785	0.01215	0.60894	Vgg_19	0.98756	0.01244	0.58042
Mob_v2_1.3	0.99119	0.00881	0.59292	Xcp_V1	0.98235	0.01765	0.70165

Listed in alphabetical order: ACC[0,1], BAC[0,1], higher is better; ERR[0,1], lower is better.

**Table 7:** Values of model geometric mean (GM), Youden’s index (YI), and discriminant power (DP) for label *lynx*.

Model	GM	YI	DP	Model	GM	YI	DP
AlxNet	0.294028	0.073167	0.448681	Mob_v2_1.4	0.486197	0.221541	0.663033
DnsNet_121	0.520312	0.264649	0.909576	Mob_v3_lrg	0.408947	0.154043	0.608400
DnsNet_161	0.508563	0.254410	0.985547	Mob_v3_lrg-min	0.371006	0.131403	0.741037
DnsNet_169	0.429780	0.179550	0.857060	Mob_v3_sml	0.304454	0.087251	0.679232
Inc_Res_v2	0.684137	0.465219	1.224305	Mob_v3_sml-min	0.265746	0.063511	0.549988
Inc_v1	0.406363	0.162545	0.995748	Ns_lrg	0.723300	0.518645	1.138357
Inc_v2	0.510974	0.253532	0.848468	Ns_mob	0.476743	0.224114	1.023565
Inc_v3	0.632676	0.393475	0.976147	Pns_lrg	0.740140	0.544287	1.209156
Inc_v4	0.690401	0.472635	1.144106	Pns_mob	0.482234	0.228639	0.978718
Mob_v1_0.25	0.216736	0.041633	0.520814	Rs_v1_101	0.418710	0.168940	0.793827
Mob_v1_0.50	0.354608	0.121370	0.804397	Rs_v1_152	0.482325	0.225044	0.819826
Mob_v1_0.75	0.399150	0.151931	0.735634	Rs_v1_50	0.385667	0.143319	0.793460
Mob_v1_1.0	0.435789	0.173473	0.586183	Rs_v2_101	0.407747	0.163196	0.956939
Mob_v2_0.35	0.315877	0.092315	0.621156	Rs_v2_152	0.497177	0.241473	0.902555
Mob_v2_0.50	0.373030	0.130956	0.678430	Rs_v2_50	0.435336	0.186680	1.006538
Mob_v2_0.75	0.417389	0.168353	0.812627	Vgg_16	0.409273	0.158574	0.702280
Mob_v2_1.0	0.473270	0.217872	0.862727	Vgg_19	0.409126	0.160845	0.776746
Mob_v2_1.3	0.435153	0.185841	0.954902	Xcp_V1	0.641665	0.403294	0.931244

Listed in alphabetical order: GM[0,1], YI[0,1], DP[0,3]; higher is better.

**Table 8:** Values of model F1 score (F1), Matthew’s correlation coefficient (MCC) and Cohen’s kappa (κ) for label *lynx*.

Model	F1	MCC	κ	Model	F1	MCC	κ
AlxNet	0.04747	0.04477	0.03969	Mob_v2_1.4	0.10162	0.11536	0.09367
DnsNet_121	0.19712	0.19935	0.19138	Mob_v3_lrg	0.08461	0.08919	0.07691
DnsNet_161	0.22783	0.22475	0.22299	Mob_v3_lrgm	0.11380	0.10976	0.10796
DnsNet_169	0.16015	0.15633	0.15481	Mob_v3_sml	0.08602	0.08077	0.08052
Inc_Res_v2	0.38796	0.39008	0.38394	Mob_v3_smlm	0.05828	0.05295	0.05206
Inc_v1	0.19251	0.19130	0.18874	Ns_lrg	0.32570	0.34747	0.32049
Inc_v2	0.16999	0.17534	0.16370	Ns_mob	0.23243	0.22826	0.22822
Inc_v3	0.23454	0.25227	0.22846	Pns_lrg	0.37306	0.38981	0.36841
Inc_v4	0.33526	0.34687	0.33042	Pns_mob	0.21765	0.21349	0.21298
Mob_v1_0.25	0.04605	0.04061	0.04059	Rs_v1_50	0.13066	0.12622	0.12518
Mob_v1_0.50	0.12489	0.11995	0.11994	Rs_v1_101	0.13780	0.13528	0.13192
Mob_v1_0.75	0.11741	0.11548	0.11115	Rs_v1_152	0.15598	0.15907	0.14967
Mob_v1_1.0	0.08000	0.08916	0.07182	Rs_v2_50	0.20925	0.20633	0.20528
Mob_v2_0.35	0.07778	0.07340	0.07146	Rs_v2_101	0.18286	0.17957	0.17873
Mob_v2_0.50	0.09864	0.09655	0.09210	Rs_v2_152	0.19074	0.19054	0.18516
Mob_v2_0.75	0.14332	0.14006	0.13766	Vgg_16	0.10942	0.10989	0.10267
Mob_v2_1.0	0.17104	0.17044	0.16529	Vgg_19	0.13089	0.12825	0.12494
Mob_v2_1.3	0.19327	0.18886	0.18884	Xcp_V1	0.20813	0.23359	0.20147

Listed in alphabetical order: F1[0,1], MCC[−1,1], κ[−1,1]; higher is better.

**Table 9:** Multi-5 confusion matrix parameter values.

TP	TN	FP *	FN *	TPR	TNR	PPV	ACC
810	291,039	935	820	0.496933	0.996798	0.464183	0.994023
ERR *	**BAC**	**GM**	**YI**	**DP**	**F1**	**MCC**	κ
0.005977	0.746865	0.703805	0.493730	1.372298	0.480000	0.477278	0.476998

*—lower values are better.

**Table 10:** Multi-4 confusion matrix parameter values.

TP	TN	FP *	FN *	TPR	TNR	PPV	ACC
772	291,314	660	858	0.473620	0.997740	0.539106	0.994830
ERR *	**BAC**	**GM**	**YI**	**DP**	**F1**	**MCC**	κ
0.005170	0.735680	0.687422	0.471359	1.433602	0.504246	0.502721	0.501658

*—lower values are better.

**Table 11:** Multi-3 confusion matrix parameter values.

TP	TN	FP *	FN *	TPR	TNR	PPV	ACC
596	291716	258	1034	0.365644	0.999116	0.697892	0.995600
ERR *	**BAC**	**GM**	**YI**	**DP**	**F1**	**MCC**	κ
0.004400	0.682380	0.604418	0.364761	1.552263	0.479871	0.503265	0.477878

*—lower values are better.

**Figure A1:**
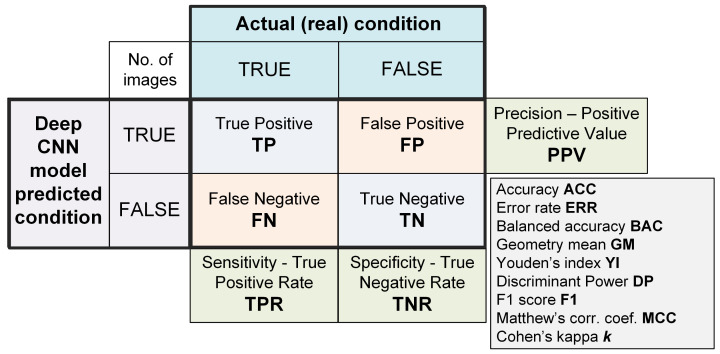
Confusion matrix structure.

## Data Availability

Image dataset and instructions to reproduce the results presented in this paper can be obtained from the following: https://vuka365.sharepoint.com/:f:/s/Lynx/Eh_oqh_eydxFiyGDU313l5QBDOyiVtVKw30jeeGAGwPG6Q?e=Nv4tNg, https://vuka365.sharepoint.com/:f:/s/Lynx/Eh_oqh_eydxFiyGDU313l5QBDOyiVtVKw30jeeGAGwPG6Q?e=Nv4tNg (accessed on 25 September 2021).

## Appendix A. Classification Models Evaluation and Metric

Binary classifier or pre-trained model, in this case, produces two predicted classes, “true” and “false”. Confusion matrix or error matrix [68] is the table that contains four outcomes produced by a binary classifier—correct positive prediction or true positive (TP), incorrect positive prediction or false positive (FP), correct negative prediction or true negative (TN), and incorrect negative prediction or false negative (FN) [69,70]. The confusion matrix structure is shown in Figure A1.

With these four parameters, additional terms are defined for a two-by-two confusion matrix [68,69,70] (Table A1). As mentioned earlier, all listed CNN models classify images collected from a camera trap. Classification results are listed in form of 1000 ImageNet classes labels and their probability. The images containing lynx were the focus of this research. In order to evaluate models’ ability to detect lynx, two aspects are important: model’s top-5 inference results and whether there is a lynx in the image. If the classifier top-5 results do list lynx for the image containing the lynx, then this is the case of a correct positive prediction or true positive (TP). If for a particular image, which depicts the lynx, the classifier top-5 results do not list a class “lynx”, it is the case of the incorrect negative prediction or false negative (FN). Furthermore, if classifier top-5 results do not list class “lynx” for the image which does not depict the lynx, it is the case of the correct negative prediction or true negative (TN). Finally, if classifier top-5 results do list the class “lynx” for the image that does not depict the lynx, it is the case of an incorrect positive prediction, or false positive (FP). From these four values, all other values (listed in the table above) are derived. These values are as follows:

1. True-positive rate (TPR) or *sensitivity*: measures the proportion of positives that are correctly identified as such (i.e., when a lynx is actually in the image, how often the classification model predicts correctly when a lynx is in the image);
2. True-negative rate (TNR) or *specificity*: measures the proportion of negatives that are correctly identified as such (i.e., when a lynx is not actually in the image, how often the classification model predicts correctly when a lynx is not in the image);
3. Positive-predictive value (PPV) or *precision*: measures the proportion of predicted positives that are actually positive (i.e., when classification model predicts the lynx in the image, or how often the predictions are correct);
4. Accuracy: the measure of the model’s correct classification;
5. Error rate: the measure of the model’s incorrect classification;
6. Balanced accuracy: used when positive and negative classes are imbalanced;
7. Geometric mean (GM): measures geometric mean of precision and sensitivity;
8. Youden’s index (YI): measures model ability to avoid misclassifications;
9. Discriminant power (DP): measures how successfully the model distinguishes between positive and negative examples;
10. F1 score: measures the harmonic mean of precision and sensitivity (or balance between precision and sensitivity);
11. Matthews correlation coefficient: correlation coefficient between the observed and predicted classifications;
12. Cohen’s Kappa: measures how well the classifier performed, compared (*Observed Accuracy*) with how well it would have performed simply by chance (*Expected Accuracy*).

The present research was performed on imbalanced data, so the selection of the appropriate evaluation model should rely on a set of different measures [71]. There are several classification metrics presented in this paper: accuracy, balanced accuracy, geometric mean, Youden’s index, discriminant power, F1 score, Matthews correlation coefficient, and Cohen’s kappa. Accuracy is not a very good measure of classification model if positive and negative classes are imbalanced, which was the case in this research; therefore, we used the balanced accuracy instead. Geometric mean, Youden’s index, and discriminant power are dependent on TPR and TNR values, and they are, similarly to balanced accuracy, suitable for imbalanced data. Geometric mean or Fowlkes–Mallows index measure the balance between classification performances in the majority and minority classes [72]. Youden’s index or bookmaker informedness measures the model’s ability to balance precision and sensitivity [72,73], while discriminant power summarizes sensitivity and specificity [73]. The F1 score is a better metric for model evaluation because accuracy does not take FP and FN into account, while F1 does. Another metric for classification model evaluation is Matthews correlation coefficient, which is considered better than the F1 score because it does not ignore TN values such as the F1 score. While accuracy highly relies on TP and TN values, the F1 score does not use TN values at all, so the classification models evaluation can be misleading. Matthew’s correlation coefficient (also called the measure of the quality of the classifications) takes into account all four values (TP, TN, FP, and FN) and “MCC is high only if your classifier is doing well on both the negative and the positive elements [74]”. According to Powers [75], MCC is a special case of Pearson’s correlation [67], so we can use the same interpretation for Phi Coefficient and MCC parameters. Cohen’s kappa uses all four values (TP, TN, FP, and FN) in order to compare classification model observed accuracy and random chance accuracy, especially if positive and negative classes are imbalanced. Cohen’s kappa coefficients are divided into several arbitrary divisions that can be used as benchmarks for result interpretation [68].

The next important subject is the interpretation of calculated values. All values derived from TP, TN, FP, and FN have values in range from 0 to 1. Values of ACC, BAC, TPR, TNR, PPV, GM, Y1, and F1 range from 0 to 1—a higher value implies better model image classification performance and vice versa. The parameter ERR also ranges from 0 to 1, but in this case, lower values are preferable. The following two mentioned parameters range from −1 to 1. The MCC value of −1 implies that actual and predicted conditions are not correlated; value 0 implies that the model is no better than accidental guessing, while value 1 implies that the model prediction capabilities are perfect. Cohen’s Kappa values also vary from −1 to 1. Values lower or equal to 0 indicate no agreement, while value 1 indicates a perfect agreement between the actual and predicted classification. Parameter DP ranges from 0 to 3. The model is a poor discriminant if DP < 1, limited discriminant if DP < 2, fair discriminant if DP < 3, and a good discriminant for all other values.

Although it is common to evaluate classification models for top-1 and top-5 results (presented in the table with the list of used image classification models), all classification models are evaluated for top-5 results in this conducted research.

**Table A1:** Confusion matrix parameters.

Parameter	Calculation
Sensitivity, Recall or True Positive Rate (TPR)	(A1) $TPR=\frac{TP}{TP+FN}$
Specificity, Selectivity or True Negative Rate (TNR)	(A2) $TNR=\frac{TN}{TN+FP}$
Precision or Positive Predictive Value (PPV)	(A3) $PPV=\frac{TP}{TP+FP}$
Accuracy (ACC)	(A4) $ACC=\frac{TP+TN}{TP+TN+FP+FN}$
Error Rate (ERR)	(A5) ERR=1−ACC
Balanced Accuracy (BAC)	(A6) $BACC=\frac{TPR+TNR}{2}$
Geometric Mean (GM)	(A7) $GM=\sqrt{TPR\cdot TNR}$
Youden’s Index (YI)	(A8) YI=TPR+TNR−1
Discriminant Power (DP)	(A9) $DP=\frac{\sqrt{3}}{\pi }\left(\log\left(\frac{TPR}{1-TPR}\right)+\log\left(\frac{TNR}{1-TNR}\right)\right)$
F1 score (F1)	(A10) $F1=\frac{2TP}{2TP+FP+FN}$
Matthews Correlation Coefficient (MCC)	(A11) $MCC=\frac{TP\cdot TN-FP\cdot FN}{\sqrt{\left(TP+FP)\cdot (TP+FN)\cdot (TN+FP)\cdot (TN+FN\right)}}$
Cohen’s Kappa (κ)	(A12) $\kappa =\frac{p_{o-p_{e}}}{1-p_{e}}$
Observed Accuracy ($p_{o}$)	(A13) $$p_{o}=\frac{TP+TN}{TP+TN+FP+FN}$$
Expected Accuracy ($p_{e}$)	(A14) $$p_{e}=\frac{\left(TN+FP)\cdot (TN+FN)+(FN+TP)\cdot (FP+TP\right)}{{\left(TP+TN+FP+FN\right)}^{2}}$$
